# Pharmaceutical efficacy of novel human-origin *Faecalibacterium prausnitzii* strains on high-fat-diet-induced obesity and associated metabolic disorders in mice

**DOI:** 10.3389/fendo.2023.1220044

**Published:** 2023-08-29

**Authors:** Meng Yang, Jing-Hua Wang, Joo-Hyun Shin, Dokyung Lee, Sang-Nam Lee, Jae-Gu Seo, Ji-Hee Shin, Young-Do Nam, Hojun Kim, Xiaomin Sun

**Affiliations:** ^1^ Department of Nephrology, Integrated Hospital of Traditional Chinese Medicine, Southern Medical University, Guangzhou, Guangdong, China; ^2^ Department of Rehabilitation Medicine of Korean Medicine, Dongguk University, Goyang-si, Republic of Korea; ^3^ Institute of Bioscience & Integrative Medicine, Daejeon University, Daejeon, Republic of Korea; ^4^ R&D Center, Enterobiome Inc., Goyang-si, Republic of Korea; ^5^ Research Group of Healthcare, Korea Food Research Institute, Wanju-gun, Republic of Korea

**Keywords:** *Faecalibacterium prausnitzii*, probiotics, anti-obesity, metabolic disorders, gut–brain axis, appetite, gut microbiota

## Abstract

**Introduction:**

Obesity and related metabolic issues are a growing global health concern. Recently, the discovery of new probiotics with anti-obesity properties has gained interest.

**Methods:**

In this study, four *Faecalibacte-rium prausnitzii* strains were isolated from healthy human feces and evaluated on a high-fat diet-induced mouse model for 12 weeks.

**Results:**

The *F. prausnitzii* strains reduced body weight gain, liver and fat weights, and calorie intake while improving lipid and glucose metabolism in the liver and adipose tissue, as evidenced by regulating lipid metabolism-associated gene expression, including ACC1, FAS, SREBP1c, leptin, and adiponectin. Moreover, the *F. prausnitzii* strains inhibited low-grade inflammation, restored gut integrity, and ameliorated hepatic function and insulin resistance. Interestingly, the *F. prausnitzii* strains modulated gut and neural hormone secretion and reduced appetite by affecting the gut-brain axis. Supplementation with *F. prausnitzii* strains noticeably changed the gut microbiota composition.

**Discussion:**

In summary, the novel isolated *F. prausnitzii* strains have therapeutic effects on obesity and associated metabolic disorders through modulation of the gut-brain axis. Additionally, the effectiveness of different strains might not be achieved through identical mechanisms. Therefore, the present findings provide a reliable clue for developing novel therapeutic probiotics against obesity and associated metabolic disorders.

## 1 Introduction

Obesity has been deemed a worldwide epidemic. In recent decades, the prevalence of overweight and obesity has steadily increased in many areas (1). World Health Organization (WHO) claims that morbid obesity develops in prolonged obese individuals, and it is associated with various metabolic disorders, such as glucose intolerance, dyslipidemia, fatty liver, hypertension, insulin resistance, glucose intolerance, and even diabetes mellitus and some cancers (2–4). Obesity is exacerbated by environmental factors, such as high-fat, high-sugar, low-fiber diet, sedentary lifestyles, sleep deprivation, abuse of antibiotics, and aging (5, 6). Nowadays, the prevention and treatment of obesity have attracted many researchers to solve the global health problem (7).

Probiotics are single or several live bacterial species which can influence gut microbial activity directly or indirectly and enhance human health (8). Over the past few years, recent evidence has shown that probiotics are safe and have rapidly become a promising natural approach for remedying metabolic-related disorders (9). Probiotics assist the host by fostering gut microbiota equilibrium and enhancing immunological diseases, inflammatory bowel disease, type 2 diabetes, and obesity, according to growing research (10–12). Several *in vivo* studies and clinical trials revealed a possible causality between probiotic consumption and obesity. For example, supplements of different *Lactobacillus* and *Bifidobacterium* strains ameliorated HFD-induced weight gain, buildup of visceral fat, insulin resistance, hepatic steatosis, and expression of various pro-inflammatory cytokines (13, 14). Except for lactic acid bacteria, more researchers are paying attention to the relationship between the next-generation anaerobic strains and metabolic diseases, e.g., *Faecalibacterium prausnitzii* (*F. prausnitzii*), *Roseburia* spp., *Akkermansia muciniphila* (15, 16). Although scientists and clinicians generally acknowledge the health benefits of probiotics, even within the same genus, different probiotic species can have varying impacts on fat buildup and obesity (17). Consequently, it is crucial to evaluate the efficacy of various probiotic strains from the same species in animal models.

According to reports, *F. prausnitzii*, which makes up more than 5% of the total bacterial population in the human gut microbiota and is one of the most prevalent anaerobic bacteria there, is a significant commensal bacterium (18). Patients with type 2 diabetes, obesity, and inflammatory bowel disease, which are characterized by food intolerance, insufficient calorie intake, and dysfunctional energy metabolism, inversely correlated with *F. prausnitzii* (19, 20). A high-fat diet (HFD) may make *F. prausnitzii* decreased. The treatment of *F. prausnitzii* in HFD-fed mice ameliorates hepatic and adipose inflammation (21, 22). Furthermore, *F. prausnitzii* abundance could increase after obese and type 2 diabetes individuals lose weight (23). Previous research has indicated that supplementary *F. prausnitzii* improves the gut permeability (24). Therefore, inflammation-related dysbiosis of the gut microbial community was treated with *F. prausnitzii* as an intervention method (25). Thus, *F. prausnitzii* may benefit human health, especially for chronic metabolic diseases.

The interactions between the brain and the gastrointestinal system are reflected in the gut-brain axis. In response to food intake, the brain gets neuronal and endocrine inputs from the gut, which are combined with signals from other organs to coordinate physiological responses (26). Many physiological processes involved in the gut–brain axis include appetite, satiety, metabolism of fat, insulin secretion/sensitivity and glucose regulation (27). The information of energy balance current state communicated from the gastrointestinal tract releases peptide hormones to the brain. Studies have documented the function of these gut hormone peptides to modulate appetite and energy expenditure through the vagus nerve and critical regions of brain activation implicated in energy homeostasis, such as the hypothalamus (26). Thus, it might be a therapeutic strategy for preventing and treating obesity.

Most comparative studies used *F. prausnitzii* type strain A2-165, which is isolated from the human intestinal tract (28). However, different strains within an identical species might initiate different host immunologic reactions (29). In the present study, a total of four novel strains of *F. prausnitzii* were initially isolated from human feces and utilized for a comparative evaluation. The *F. prausnitzii* type strain A2-165 and Orlistat were used as positive controls. The selected strains were employed to evaluate their efficacy in treating obese mice induced by a HFD, with the aim of establishing foundational evidence for potential future applications of *F. prausnitzii*.

## 2 Materials and methods

### 2.1 Bacterial strains and growth conditions

All of the *F. prausnitzii* was isolated from feces collected from healthy Koreans after obtaining informed consent from each subject. The genetic diversity of isolated *F. prausnitzii* were analyzed, and the detailed results are presented in **Supplementary Section** (**Figure S1**). This research was approved by the Institutional Review Board of Dongguk University, Ilsan Hospital (IRB# 2018-06-001-012). The reference strain A2-165 (Deutsche Sammlung von Mikroorganismen [DSM] 17677) was obtained from the Leibniz-Institute DSMZ-German Collection of Microorganism and Cell Cultures). The extremely oxygen-sensitive bacteria were grown in brain-heart infusion medium supplemented with 0.5% (w/v) yeast extract, 0.1% (w/v) cellobiose, 0.1% (w/v) maltose, and 0.05% (w/v) L-cysteine, at 37°C in an anaerobic chamber (atmosphere of 5% CO2, 5% H2, and 90% N2). 16S rRNA gene sequencing for the identification of *F. prausnitzii* was performed after polymerase chain reaction (PCR) amplification of a region of the 16S rRNA gene. For the animal experiments, all of the *F. prausnitzii* strains were cultured anaerobically in a soy-peptone based medium with some supplements and centrifuged at 12,000 × g for 5 min. They were then adjusted to an end concentration of 1 × 10^8^ CFU/150 µl using anaerobic PBS with 20% glycerol and stored at −80°C until use.

### 2.2 Animals and treatments

Seven-week-old male C57BL/6 mice were obtained from Orient Bio (Seongnam, South Korea). The mice were housed under controlled conditions (a 12:12-hr light-dark cycle, temperature of 20 ± 3°C, and humidity 55 ± 5%) with ad libitum access to water and a standard chow diet (FeedLab, Guri, South Korea) for 10 days of acclimatization. All experiments were approved by the institutional animal care and use committee (IACUC) of the Dongguk University (2020-11208) and conducted according to the guidelines of the National Rearch Council (Guide for the Care and Use of Laboratory Animals, 2011). Diets were purchased from Reseach Diets, Inc. (Nwe Brunswick, NJ, USA): normal diet (Nor) containing 10% calories from the fat with 3.85 kcal/g (19.2% protein, 67.3% carbohydrate, and 4.8% fat) and HFD containing 60% calories from the fat with 5.24 kcal/g (26.2% protein, 26.3% carbohydrate, and 34.9% fat).

After acclimatization, 72 mice were weighted and randomly divided into eight groups (n = 9) as follows: (1) Nor group; (2) HFD group; (3) Orilistat-treated HFD group (XEN); (4) *F. prausnitzii* type strain A2-165-treated HFD group (A2-165); (5) *F. prausnitzii* EB-FPDK3-treated HFD group (DK3); (6) *F. prausnitzii* EB-FPDK6-treated HFD group (DK9); (7) *F. prausnitzii* EB-FPDK11-treated HFD group (DK11); (8) *F. prausnitzii* EB-FPYYK1-treated HFD group (YK1). Mice had ad libitum access to diets and sterile water. Orlistat (Xenical®; Roche, Basel, Switzerland) was dissolved in sterile PBS at pH 7.4 and administrated orally to the Xen group (10 mg/kg/day). The live bacterial samples were resuspended in sterile PBS and then freshly administrated to the A2-165, DK3, DK9, DK11, and YK1 groups via oral gavage in sterile PBS with 20% glycerol at a dose of 1 × 10^8^ CFU/150 µl per animal. The mice in the NOR and HFD groups were given only sterile PBS with 20% glycerol as a vehicle. The treatments were carried out 6 days per week for 12 weeks. The body weight of mice was assessed weekly, and food intake was recorded three times a week. Fecal samples were collected at weeks 0 and 12 post-treatment and kept at -80°C for further microbiome analysis

At the end of the experiment, mice were fasted for 12 h and blood was collected by cardiac puncture under anesthesia with a mixture of tiletamine-zolazepam (Zoletil 50, Virbac, Carros, France). Serum was separated by centrifuging at 2,000 × g for 15 min at 4°C and kept at −80°C until further biochemical analyses. The spleen, liver, large intestine, small intestine, and adipose tissues was removed, washed with ice-cold PBS, dried, and then weighed. Portions of the liver, large intestine, small intestine, adipose, and hypothalamus were removed and flash-frozen in the liquid nitrogen and stored at −80°C until further use. The other portions of the tissues were fixed with 4% paraformaldehyde (PFA) (Junsei, Tokyo, Japan) overnight at 4°C and then embedded in paraffin.

### 2.3 Serum biochemical analyses

Serum samples were analyzed for total cholesterol (TC), triglyceride (TG), alanine aminotransferase (ALT), and aspartate aminotransferase (AST) using commercial colorimetric assay kits (Asan Pham. Co., Seoul, South Korea). Insulin levels were determined using an enzyme-linked immunosorbent assay (ELISA) kit (Morinaga, Yokohama, Japan). Insulin resistance was estimated by homeostasis model assessment of insulin resistance (HOMA-IR) index calculated as follows: HOMA-IR = [(fasting glucose (mg/dL) x fasting insulin (μU/mL))/405. Lower HOMA-IR values indicated better insulin sensitivity and vice versa.

### 2.4 Oral glucose tolerance test

Mice were fasted for 14h with free access to drinking water at week 11 of study period and administrated with glucose solution (2 g/kg body weight) by oral gavage. Blood glucose level was measured from the tail vein using an Accu-Chek Active blood glucose meter (Roche Diagnostics Corp, Rotkreuz, Switzerland) at 0, 30, 60, 90, and 120 min after injection. The glucose area under the curves (AUC) during the OGTT were calculated using GraphPad Prism 5.0 software (San Diego, CA, USA).

### 2.5 Histology analysis

PFA-fixed paraffin-embedded sections (4 μm) of the liver, intestine and mesenteric adipose tissues were placed on positively charged glass slides, de-waxed in xylene (Sigma Chemicals, St Louis, MO, USA), dehydrated in a gradual ethanol series, and stained with either hematoxylin and eosin (Sigma-Aldrich, St. Louis, MO, USA), Alcian blue (AB) solution (Abcam, Cambridge, UK) for goblet cells, or Oil Red O (Sigma-Aldrich) for fatty liver tissues according to the manufacturer’s protocol. Tissues were examined under an inverted light microscope (Olympus, Tokyo, Japan). Representative images of the liver and mesenteric adipose tissue were taken from three individual liver and mesenteric adipose samples in each group at 4 x and 20 x magnifications, while for intestine AB stain, representative images were taken from six to seven individual samples in each group. The liver steatosis area and the Oil Red O-stained area were assessed on the images taken with Image-Pro Plus 6.0 software. The AB-positive area, villus length, and the number of the goblet cells were determined from image J software via images. The AB-positive goblet cells were quantified by counting the positive cells per crypt in all crypts per colon section, three sections per mouse. The villus length was measured from the base of the villus to the top in Five to six villi in section, three sections per mouse.

### 2.6 Fecal bacterial DNA extraction and sequencing analysis

DNA from fecal samples was extracted by the QIAamp® DNA Stool Mini Kit (QIAGEN, Hilden, Germany) as previously described (30). The V1–V2 16S rRNA hypervariable regions were amplified using the Bio-Rad C1000 Touch thermal cycler (Hercules, CA, USA) with primers containing a unique 10-base barcode to tag each PCR product. The PCR amplicons were purified using the QIAquick PCR purification kit (QIAGEN, Hilden, Germany) and then sequenced using the Ion Torrent PGM system (Thermo Scientific, DE, USA). Sequences below 300 bp in length and low-quality score below 20 were removed. All effective sequences were clustered into operational taxonomic units (OTUs) using SILVA rRNA gene database (http://www.arb-silva.de) with a threshold of 97% sequence identity. The Quantitative Insights into Microbial Ecology (QIIME) software was used to select the representative reads of each OTU and to calculate beta diversity. The clustering pattern of the microbial composition was presented by principal coordinated analysis (PCA) using UniFrac distance matrices. The differences between groups in the taxa with varying abundances were sassed by using the linear discriminant analysis (LDA) effect size (LEfSe). In LEfSe, a size-effect threshold of 2.0 on the logarithmic linear discriminant analysis (LDA) score and an alpha value of 0.05 for the factorial Kruskal-Wallis test among the classes were used. The microbiota sequencing data have been deposited to the NCBI Sequence Read Archive (SRA) database under PRJNA885263. SPSS software (version 19.0) was used to assess the relationship strength between relative abundance and obesity-related parameters by the two-tailed Pearson’s correlation test.

### 2.7 RNA extraction and real-time PCR

Total RNA form liver, adipose, colon, jejunum, and hypothalamus tissues were isolated with TRIsureTM reagent (Meridian Life Science Inc, TN, USA) following the manufacturer’s protocol. The A260/280 ratio confirmed RNA purity. One μg of total RNA was reverse-transcribed with an AccuPower RT PreMix with oligo-(dT)18 primer (Bioneer, Daejeon, South Korea). Quantitative real-time PCR was performed using the LightCycler® 480 Real-Time PCR. System (Roche, Mannheim, Germany) and SYBR® Green real-time PCR kit (Toyobo, Tokyo, Japan) according to the manufacturer’s instruction. The GAPDH gene was used as a reference. The sequences of primers were shown in the **Supplementary Table** (**Table S1**) in the Online Repository.

### 2.8 Statistical analyses

All data are presented as means ± standard error of the means (SEM). GraphPad Prism 8.0 software (San Diego, CA, USA) was used to determine the statistical significance. Data were analyzed by an unpaired two-tailed Student’s t-test for a two-group comparison or one-way ANOVA with Bonferroni-corrected *post hoc* tests for multiple comparisons unless stated otherwise. P-values less than 0.05 was considered statistically significant.

## 3 Results

### 3.1 Effect of *F. prausnitzii* administration on body weight gain, fat mass, and calories intake *in vivo*

**Figure 1:**
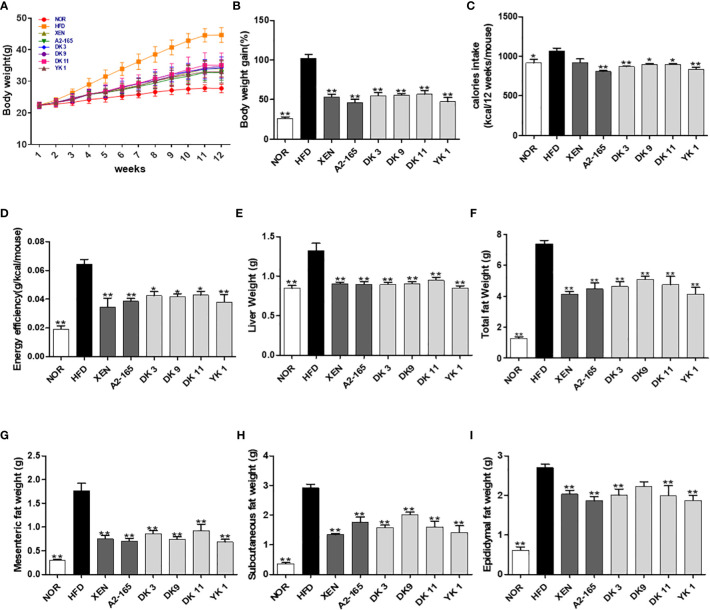
Effect of *F. prausnitzii* on the body weight, adiposity, organ weight, and caloric intake in mice. **(A)** Body weight measured weekly. **(B)** Total body weight gain. **(C)** The averaged calorie intake of each mouse and **(D)** energy efficiency (body weight gain/food intake). **(E)** Liver weight. **(F)** Representative weight of total fat (subcutaneous fat+epididymal fat+mesenteric fat), mesenteric fat **(G)**, subcutaneous fat **(H)**, and epididymal fat **(I)**. Data are represented as the mean ± SEM (n=9). The statistics were analyzed by one-way ANOVA. *p<0.05 and **p<0.01 versus the HFD group.

Mice fed a 12-week high-fat-diet showed a 2.9 times increase in body weight gain (p < 0.01), 17% increase in calorie intake (p < 0.05), and 2.35 times increase in energy efficiency (p < 0.01) compared to the normal diet group (**Figures 1A–C**). However, the body weight gain was markedly reduced by the microbial strains compared to HFD, with reduced rates of A2-165: 40%; DK3: 37%; DK9: 31%; DK11: 36%; YK1: 44%; XEN: 44% (p < 0.05 or p < 0.01, **Figure 1B**). Furthermore, all these treated groups showed a significant decrease in calories intake compared to the HFD group (**Figure 1D**). The increased liver weights by HFD feeding also resulted in significant reductions in the other treatment groups (**Figure 1E**). The total visceral fat mass weight of the HFD mice (**Figure 1F**) was 4.79 times greater than the normal group (p < 0.01). This effect was explained mainly by a reduction in mesenteric and subcutaneous fat upon treatment with all four selected pasteurized *F. prausnitzi*i strains: DK3, DK9, DK11, and YK1. By contrast, only DK3, DK11 and YK1 reduced the epididymal fat significantly (**Figures 1G–I**). In addition, the relative liver and epididymal weights did not show a significant difference among all groups, while other the tissue mass as relative to body weight also showed a similar pattern with absolute organ mass (**Figure S2**).

### 3.2 *F. prausnitzii* prevented obesity and improved glucose homeostasis in HFD-fed mice

**Figure 2:**
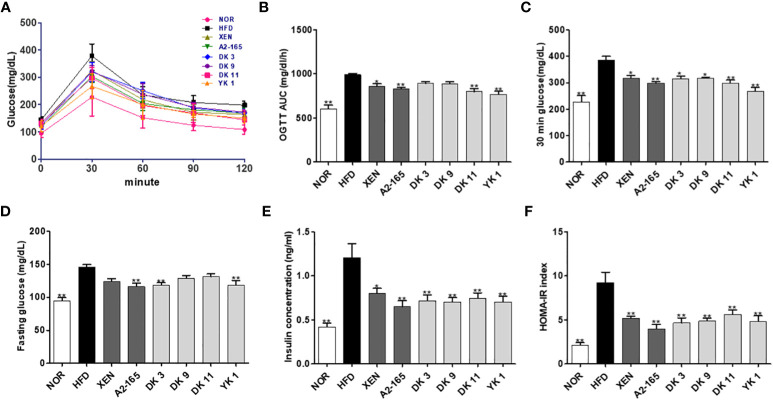
*F. prausnitzii* strains improved glucose homeostasis in HFD-induced obese mice. **(A)** Curve of oral glucose tolerance tests (OGTT). **(B)** Areas under the curve (AUC) of OGTT measured between 0 and 120min after glucose administration. **(C)** 30min glucose. **(D)** Fasting glucose. **(E)** Serum insulin and **(F)** HOMA-IR. Data are represented as the mean ± SEM (n=9). The statistics were analyzed by one-way ANOVA. *p<0.05 and **p<0.01 versus the HFD group.

The impact of the *F. prausnitzii* strains treatment on glucose homeostasis and insulin sensitivity in mice was determined by oral glucose tolerance tests (OGTT) (**Figure 2A**). HFD-fed mice showed the 54% higher in fasting glucose level (p < 0.01), the 69% higher in 30 minutes’ glucose level (p < 0.01), and the 64% higher in the OGTT area under the curve (AUC) (p < 0.01) compared with normal low-fat diet food mice (**Figures 2B–D**). The fasting glucose level was changed significantly by the strains A2-165, DK3, and YK1 treatment with reduction rate 20%, 19%, 19% respectively (p < 0.01), while other treatment groups showed a slight reduction compared to the HFD group (**Figure 2D**). The oral gavage glucose solution at 30 minutes recorded the highest glucose level. The HFD group had a significantly higher level than the other groups, which was decreased significantly by all treatments, with the decrease rates of XEN:18%; A2-165%; DK3:18%; DK9:18%; DK11:23%; YK1:31% (p < 0.05 or p < 0.01) (**Figure 2C**). In the OGTT, the AUC values also showed upregulation by HFD feeding compared to the normal group, while it was reduced significantly by treatment with XEN, A2-165, DK11, and YK1, with decrease rates of 13%, 16%, 19%, 23% respectively (p < 0.05 or p < 0.01); the other treatment groups showed a slight decrease compared to HFD group (**Figure 2B**). The serum insulin concentration was elevated significantly in response to HFD feeding, while it was reduced markedly by the microbial strains and XEN treatment, with reduction rate as XEN: 33%; A2-165: 46%; DK3: 41%; DK9: 42%; DK11: 38%; YK1: 42% (p < 0.05 or p < 0.01) (**Figure 2E**). Homeostatic model assessment for insulin resistance index (HOMA-IR) showed a significant increase in the HFD group and a noticeable decrease in the treatment groups compared to the HFD group, with decrease rates as XEN:44%; A2-165:57%; DK3:49%; DK9:47%; DK11:39%; YK1:47% (p<0.01) (**Figure 2F**).

### 3.3 *F. prausnitzii* administration improved the lipid metabolism parameter of HFD mice

The obesity-associated serum biomarkers were further analyzed to investigate lipid metabolism in response to diet in HFD mice (**Table 1**). The total cholesterol (TC), triglyceride (TG), aspartate aminotransferase (AST), and alanine aminotransferase (ALT) were increased significantly in the HFD group than in the normal group (p < 0.05 or p < 0.01). Moreover, the levels of TC all showed downward trends after treatment. Among them, the XEN and YK1 treatments caused a 22% and 28% reduction in TC levels compared to the HFD group, respectively (p < 0.05 or p < 0.01). In addition, the TG level showed similar results to the TC. The serum TG could be improved by all treatment ways and compared with the HFD group. The YK1 strain reduced the TG level by 42% compared to the HFD group (p < 0.01). All treatment groups could significantly improve the serum AST and ALT levels (p < 0.05 or p < 0.01), which are biomarkers of the hepatic function. These results showed that *F. prausnitzii* administration improved the lipid metabolism in serum.

**Table 1:** *F. prausnitzii* strains improved lipid metabolism parameter of the HFD mice.

Contents		NOR	HFD	XEN	A2-165	DK3	DK9	DK11	YK1
Serum	TC (mg/dL)	108.0 ± 17.63**	217.7 ± 9.45	170.0 ± 9.42**	175.6 ± 3.76**	182.0 ± 7.56*	182.7 ± 6.29**	189.7 ± 4.51*	157.8 ± 8.75**
	TG (mg/dL)	33.6 ± 4.71**	72.8 ± 10.86	58.0 ± 4.57	56.9 ± 6.53	60.9 ± 7.34	51.7 ± 4.69	56.6 ± 3.83*	42.56 ± 2.83*
	AST (IU/L)	33.9 ± 3.84*	52.3 ± 4.57	29.5 ± 3.53**	26.3 ± 4.66**	31.0 ± 4.61**	28.3 ± 2.04**	24.6 ± 2.42**	21.1 ± 1.43**
	ALT (IU/L)	9.1 ± 1.56*	17.3 ± 2.44**	7.2 ± 0.78**	7.9 ± 1.57**	8.8 ± 1.18**	8.2 ± 1.02**	9.4 ± 1.34**	5.1 ± 2.14**

The values are presented as the mean ± SEM. *p<0.05, **p<0.01 versus the HFD group; HFD, high-fat diet; AST, aspartate aminotransferase; ALT, alanine aminotransferase; TG, triglyceride; TC, total cholesterol.

### 3.4 *F. prausnitzii* administration reversed the HFD-induced effects on liver damage

**Figure 3:**
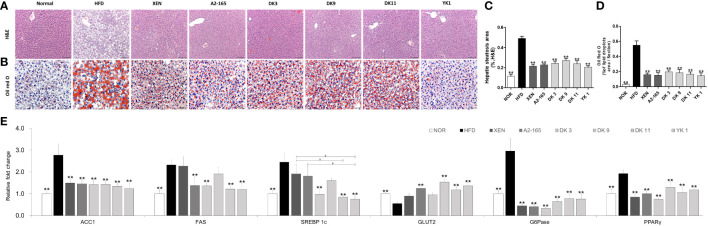
*F. prausnitzii* administration alleviated hepatic steatosis and improved hepatic function in mice. **(A)** Histological analysis of H&E stain on liver sections of mice (n=3 per condition, scale bar, 100μm). **(B)** Representative Oil Red O staining for fat deposition measurement in liver. **(C)** The liver steatosis area. The liver steatosis was characterized by micro- and macrovacuolization. **(D)** Oil Red O-stained fat deposition area. (n=3 per condition, scale bar, 50μm). Representative mRNA expression of **(E)** ACC1, FAS, SREBP1c, GLUT2, G6Pase, and PPARγ in liver tissue from mice (n=9 mice/group). Data are represented as mean ± SEM. The statistics were analyzed by one-way ANOVA. **p<0.01 versus the HFD group. ^#^p<0.05.

For requirements for a specific article type please refer to the Article Types on any Frontiers journal page. Because the hepatic function is associated with HFD- induced obesity (31), the effects of *F. prausnitzii* supplement on HFD-induced hepatic function were next determined. Histological analysis of H&E staining and lipid staining on the liver sections (**Figures 3A–D**) confirmed the normal histology in normal low-fat diet-fed mice. In contrast, mice after 12 weeks of HFD developed a fatty liver phenotype, featuring a pale liver appearance caused by extensive fat accumulation, including cases of both macrovesicular and microvesicular steatosis. As expected, treatment with DK3, DK9, DK11, and YK1 resulted in markedly reduced vacuolation. The XEN and A2-165 groups also showed a slight improvement in fat accumulation and hepatic steatosis (**Figures 3A, C**). Moreover, the Oil Red O examination showed that the HFD-fed mice had 16.77 times more lipid droplets in the liver tissues compared to the normal group (p < 0.01). As shown in **Figures 3B, D**, the treatment groups showed obvious down-regulation of the lipid droplets compared to the HFD group, with reduction rates as XEN: 71%; A2-165: 72%; DK3: 64%; DK9: 66%; DK11: 70%; YK1: 73% (p < 0.01). In addition, the hepatic gene expression of ACC1, FAS, and SREBP1c in the normal group was significantly lower than in the HFD group (p < 0.01). In contrast, the expression of ACC1 mRNA was decreased significantly in the liver of the HFD-fed animals treated with all four selected pasteurized *F. prausnitzii* strains (p < 0.01). From the treatment, except DK9, other selected strains all significantly decreased the FAS and SREBP1c (p < 0.01). Among them, the hepatic expression of SREBP1c was significantly lower in the DK11 and YK1 groups than in the XEN group (p < 0.05) (**Figure 3E**).

On the other hand, the gene expression of (GLUT2), Glucose 6-phosphatase(G6Pase), and peroxisome proliferator-activated receptor-gamma (PPARγ) are associated with gluconeogenesis, but only G6Pase and PPARγ showed significant changes compared to the normal and HFD groups (**Figure 3E**). By treatment, in addition to DK3 and XEN, the other treatment methods upregulated the GLUT2 level markedly, but all treatments could inhibit G6Pase and PPARγ expression significantly. Consistent with the decrease in liver weight, the improvement of hepatic steatosis in histochemical and qPCR analysis showed that the *F. prausnitzii* treatment could ameliorate the hepatic steatosis and damage caused by the HFD and improve the hepatic function in mice.

### 3.5 Effect of *F. prausnitzii* administration on the adipokine profile of adipose tissue

For requirements for a specific article type please refer to the Article Types on any Frontiers journal page. Adipocyte hypertrophy is the major mechanism for the expansion of adipose tissue during the obesity development (32). To investigate the effects of *F. prausnitzii* administration on lipid accumulation in the adipose tissue of HFD-fed mice, three different parts of adipose tissue samples were collected from the mice and the amount of fat was assessed. The fat mass weight and adipocytes size of mesenteric fat was examined by H&E staining.

**Figure 4:**
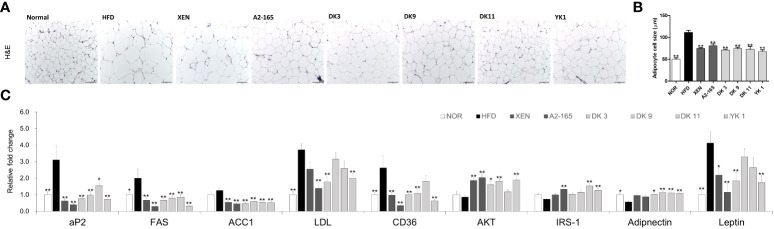
Effects of *F. prausnitzii* on adipokine profile of adipose tissue in HFD-fed mice. **(A)** Histological analysis (H&E staining) of sections of mesenteric fat tissues (n=3 per condition, scale bar, 100μm). **(B)** The average diameters of adipocytes in randomly chosen fields were measured and presented as pixels using Image-Pro Plus 6.0. **(C)** Ap2, FAS, ACC1, LDL, CD36, AKT, IRS-1, Adiponectin, and Leptin mRNA levels in adipose tissues (n=9). Data are represented as the mean ± SEM. The statistics were analyzed by one-way ANOVA or Student’s t-test. *p<0.05 and **p<0.01 versus the HFD group.

As shown in the H&E staining pictures (**Figure 4A**), the results revealed up-regulation in the HFD group than the normal group of lipid accumulation in the mesenteric fat and a significant increase in the average fat cell size (p < 0.01). After treatment with different supplements, however, the fat cell size decreased significantly compared to the HFD group, with the reduction rates as XEN: 32%; A2-165: 27%; DK3: 36%; DK9: 33%; DK11: 35%; YK1: 39% (p < 0.05 or p < 0.01) (**Figure 4B**).

Obesity leads to adipose tissue dysfunction and vice versa (33). The expression levels of genes associated with lipogenesis or adipogenesis in mesenteric fat tissue were measured by RT-PCR to clarify the mechanism of the *F. prausnitzii* strains on HFD-induced obesity (**Figure 4C**). The expression levels of CD36 (p < 0.01), FAS (p < 0.05), and LDL (p < 0.01) mRNAs increased significantly after HFD feeding compared to the low-fat feeding group (normal group), but ACC1 showed an increasing trend in the HFD group. However, FAS and ACC1 levels were markedly reversed after all ways of treatments (p < 0.01). The selected strains, DK3 (p < 0.01), DK9 (p < 0.01), and YK1 (p < 0.01), resulted in markedly reduced CD36 mRNA expression, and DK3 (p < 0.01) and YK1 (p < 0.01) produced a significant decrease in the LDL levels. In addition, after the different treatments, the levels of the glucose and lipid metabolism regulation marker (aP2 and AKT), insulin-related markers (Adiponectin and IRS-1), and leptin, a hormone that facilitates the regulation of feeding and energy homeostasis (**Figure 4C**). A definite, insignificantly lower AKT, IRS-1, and Adiponectin mRNA levels were observed in the HFD groups compared to the normal groups. In contrast, the aP2 (p < 0.01) and leptin (p < 0.01) genes showed significantly higher expressions in the HFD group than in the normal group. After treatment, the mRNA levels of aP2 decreased (p<0.05 or p<0.01)., and except for the DK9 and DK11 groups, the leptin (p < 0.01) level also decreased significantly. Treatment with all four selected strains in the HFD groups, but not the other treatments, upregulated the expression of the Adiponectin gene significantly (p < 0.05 or p < 0.01). All the treatments except DK11 could increase the levels of AKT (p < 0.05 or p < 0.01), and the type strain A2-165 (p < 0.01), DK11 (p < 0.01), and YK1 (p < 0.01) could upregulate the IRS-1 mRNA expression level. Therefore, *F. prausnitzii* affects the adipose tissue metabolism.

### 3.6 *F. prausnitzii* treatment exerted anti-inflammatory effects in HFD-fed mice

**Figure 5:**
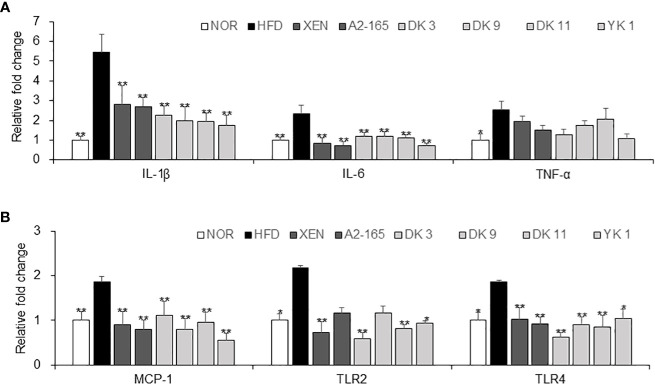
Anti-inflammatory effects of *F. prausnitzii* in the colon tissue. mRNA levels of inflammatory cytokines. **(A)** IL-1β, IL-6, and TNF-α, **(B)** MCP-1, TLR-2, and TLR4 in the colon tissue of each group were determined by real-time PCR. Data are represented as the mean ± SEM. The statistics were analyzed by one-way ANOVA or Student’s t-test. *p<0.05 and **p<0.01 versus the HFD group.

The protective activity of pasteurized *F. prausnitzii* against HFD-induced inflammation in the colon was evaluated. This study examined the effects of *F. prausnitzii* in the HFD group on the gene expression of the following, which play vital roles in the inflammation and inflammatory signaling pathways: pro-inflammatory cytokines IL-1β, IL-6, and TNF-α; pro-inflammatory chemokine MCP-1; toll-like receptors TLR2 and TLR4, (**Figures 5A, B**). The colonic mRNA levels of IL-1β (p < 0.01), IL-6 (p < 0.01), TNF-α (p < 0.05), MCP-1 (p < 0.01), TLR2 (p < 0.05), and TLR4 (p < 0.05) were significantly higher in the HFD group than in the normal group. In particular, exposure of the HFD mice to all *F. prausnitzii* strains depleted the expression of IL-1β, IL-6, MCP-1, and TLR4 and suppressed the expression of the TNF-α gene in the colonic tissue, even though the latter change was insignificant. Treatment of the HFD-fed animals with XEN (p < 0.01), DK3 (p < 0.01), DK11 (p < 0.01), and YK1 (p < 0.05), but not the other treatments, decreased the expression of the TLR2 gene significantly.

### 3.7 *F. prausnitzii* treatment improved the intestinal barrier function in HFD-fed mice

**Figure 6:**
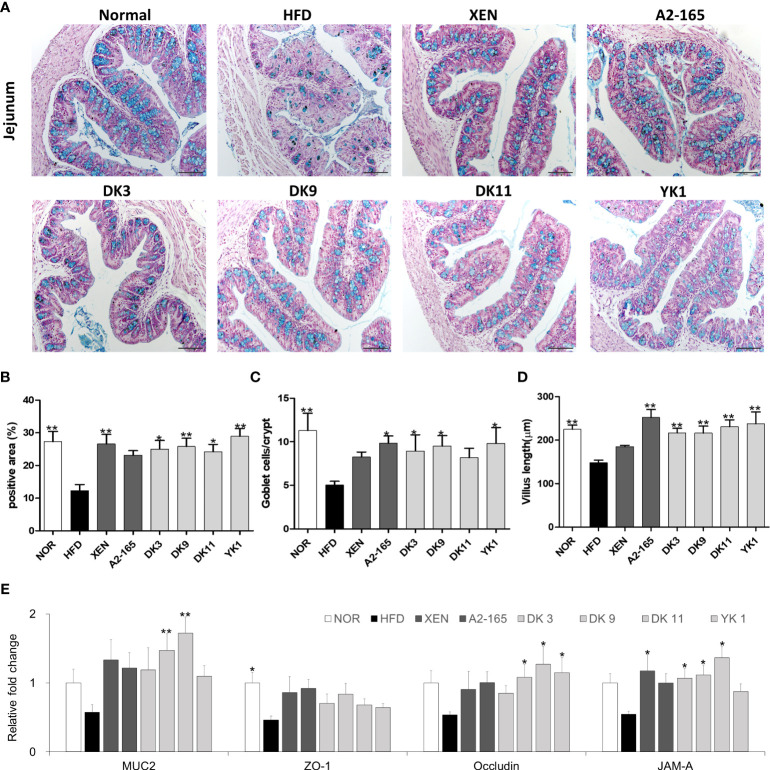
*F. prausnitzii* improved the functions and integrity of jejunum in HFD-fed mice. **(A)** Representative microscopic images demonstrating Alcian blue (AB)-staining of colonic tissue sections of mice from different experimental groups at a magnification of 200×, **(B)** the proportion of AB-positive area (%), **(C)** number of goblet cells, and **(D)** length of the villus in colonic tissue sections (n=3 per condition, scale bar, 100μm). Representative mRNA expression of **(E)** Muc2, ZO-1, Occludin, and JAM-A in intestine tissue from mice (n=9 mice/group). The statistics were analyzed by one-way ANOVA. *p<0.05 and **p<0.01 versus the HFD group.

Previously studies suggested that HFD feeding induced various dysbiosis of the intestines, which is associated with physiopathological changes, such as damaged mucus production and secretion, and injured the gut integrity and permeability of the intestinal epithelium (34). The intestine tissue was observed by AB staining to determine the effects of *F. prausnitzii* on the intestinal structure. As expected, HFD - induced obesity mice resulted in marked changes in the intestinal architecture (**Figure 6A**). In particular, a 55% decrease in the AB-stained area was observed in the HFD group compared with the normal group (p < 0.01), which is consistent with these results. The number of goblet cells (p < 0.01) and villus length (p < 0.01) were both lower than the normal group (**Figures 6B–D**). Nevertheless, the AB-positive area that represents the acidic mucins was improved markedly in four selected *F. prausnitzii* strain groups (p < 0.05 or p < 0.01) (**Figure 6B**). In support of the above results, a significant increase in the villus length was observed after treatment with A2-165 and all four selected strains compared to HFD control, with the increase rate as A2-165: 70%; DK3: 46%; DK9: 46%; DK11: 56%; YK1: 60% (p < 0.05 p < 0.01) (**Figure 6D**).

The *F. prausnitzii* supplement improved the mRNA expression of the intestine tissue and gut integrity (**Figure 6E**). Tight junctions connect the epithelial cells and play a key role in regulating the intestinal-barrier functionality (35). This study examined the expression levels of colonic mucin 2 (Muc2), ZO-1, Occludin, and JAM-A mRNAs in the intestine tissue by qRT-PCR. Compared to the untreated group (normal group), the mRNA expression of ZO-1 was inhibited significantly by HFD-feeding (p < 0.05). On the other hand, treatment of the HFD group with the XEN (p < 0.05), DK3 (p < 0.05), DK9 (p < 0.05), and DK11 (p < 0.05), but not A2-165 and YK1, increased the mRNA level of JAM-A significantly. The gene expression of occludin was upregulated significantly in the HFD group after treatment with DK9 (p < 0.05), DK11 (p < 0.05), and YK1 (p < 0.05) strains, but not the other regimens. Moreover, the gene expression of Muc2 was upregulated significantly by the oral DK9 (p < 0.01) and DK11 (p < 0.01) strains in the HFD-fed mice.

### 3.8 *F. prausnitzii* modulated hormone secretion and regulated appetite by affecting the gut–brain axis

**Figure 7:**
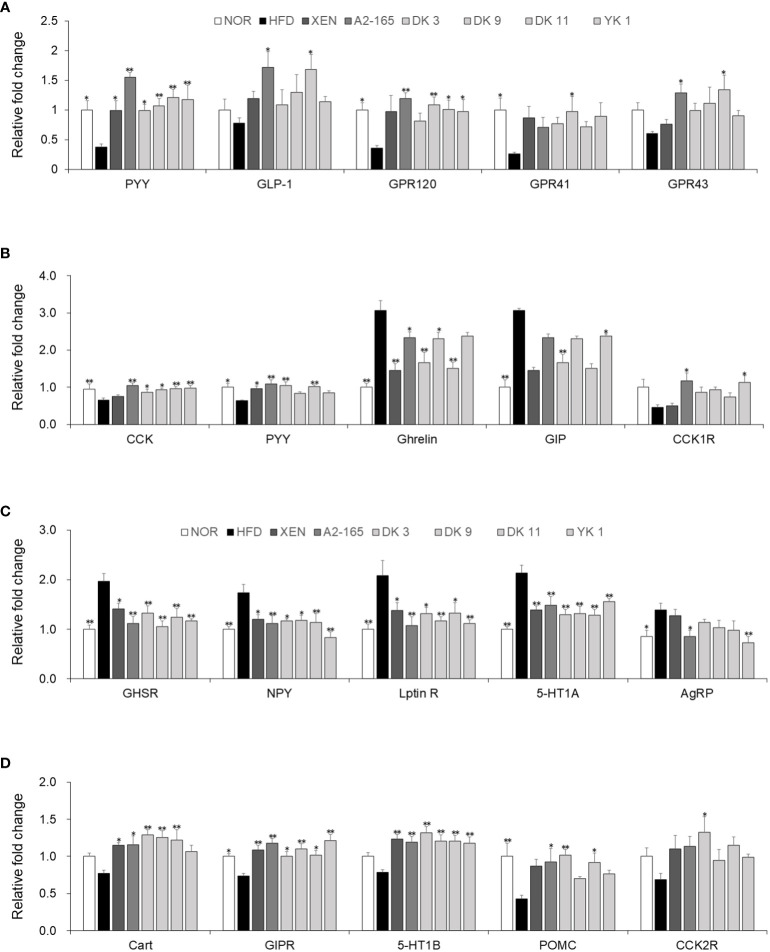
*F. prausnitzii* modulated hormone secretion and regulated appetite in HFD-fed mice. **(A)** PYY, GLP-1, GPR120, GPR41, and GPR43 mRNA levels in colonic tissues (n=9). **(B)** CCK, PYY, Ghrelin, GIP, and CCK1R mRNA levels in jejunum tissues (n=9). Representative mRNA expression of **(C)** GHSR, NPY, Leptin R, 5-HT1A, and AgRP and **(D)** Cart, GIPR, 5-HT1B, POMC, and CCK2R in hypothalamus tissue from mice (n=9 mice/group). The statistics were analyzed by one-way ANOVA. *p<0.05 and **p<0.01 versus the HFD group.

For requirements for a specific article type please refer to the Article Types on any Frontiers journal page. The gut–brain axis reflects the interactions between the gastrointestinal system and the brain in general. The brain receives both neural and endocrine inputs from the gut in response to food intake, which is integrated with signals from other organs to orchestrate physiological responses (26). Major integrating centers within the brain are the hypothalamic nuclei. Compared with the HFD-fed mice, food intake was decreased significantly by the *F. prausnitzii* strains, with the reduction rates as A2-165: 24%; DK3: 18%; DK9: 16%; DK11: 16%; YK1: 22%, (p < 0.05 or p < 0.01) (**Figure S3**). In this study, the hypothalamus, colonic, and small intestine tissue were used to provide evidence of gut-brain cross-talk involved in regulating food intake. Compared to the normal low-fat diet group, the gene expression of PYY (p<0.05), GPR120 (p<0.05), and GPR41 (p<0.05) of colonic tissues was markedly lower in the HFD group in response to feeding HFD. On the other hand, treatment of the HFD-fed animals with all the *F. prausnitzii* strains and XEN elevated the mRNA level of PYY significantly (p < 0.05 or p < 0.01). Similar to PYY, except for DK3, the other treatments could increase the GPR120 mRNA levels significantly (p < 0.05 or p < 0.01). Exposing the HFD group to type strain A2-165 and DK11, but not other treatments, upregulated the expression of the GLP-1 (p < 0.05) and GPR43 (p < 0.05) genes significantly. After treatment with DK9 (p < 0.05), the mRNA levels of GPR41 were also upregulated markedly compared to the HFD group (**Figure 7A**). In addition, the small intestine mRNA levels of CCK (p < 0.01) and PYY (p < 0.05) were significantly lower in the HFD group than in the normal group. In contrast, the gene expression of GIP (p < 0.01) and ghrelin (p < 0.01) was significantly higher in the HFD group than in the normal group. Exposure of the HFD group to all *F. prausnitzii* strains upregulated the mRNA level of CCK markedly (p < 0.05 or p < 0.01). In contrast, the treatment of the HFD-fed mice with the *F. prausnitzii* strains except YK1 depleted the expression of Ghrelin genes significantly (p < 0.05 or p < 0.01). The small intestine expression of the PYY gene in the HFD group was upregulated significantly by XEN, A2-165, DK3, and DK11 (p < 0.05 or p < 0.01). Treatment of HFD-fed mice with A2-165 and YK1, but not other treatments, increased the CCK1R (p < 0.05) gene expression, DK3 and YK1 increased the GIP (p<0.01) gene expression compared with HFD group (**Figure 7B**). Furthermore, the *F. prausnitzii* supplement modulated the appetite-related hormone mRNA expression in the hypothalamus (**Figures 7C, D**).

The gene expression levels of GHSR, NPY, Leptin R, 5-HT1A, and AgRP in the hypothalamus were significantly higher in the HFD group than in the normal group (p < 0.05 or p < 0.01). On the other hand, treatment of the HFD group with XEN and the *F. prausnitzii* strains decreased the mRNA levels of GHSR, NPY, Leptin R, and 5-HT1A (p < 0.05 or p < 0.01). The gene expression of AgRP was down regulated significantly in the HFD group upon treatment with type train A2-165 (p < 0.05) and YK1 (p < 0.01), but not the other regimens. Significantly lower expression of the GIPR (p < 0.05) and POMC (p < 0.01) genes was observed in the hypothalamus of the HFD group vs. the normal group. A definite but insignificantly higher mRNA levels of Cart, 5-HT1B, and CCK2R were observed in the HFD group than in the normal group. The mRNA levels of GIPR (p < 0.05 or p < 0.01) and 5-HT1B (p<0.05) were increased significantly in the hypothalamus of the HFD-fed mice by all treatment ways. Cart gene expression in the HFD-fed mice was also upregulated significantly upon treatment with XEN and F. prausnitzii strains, except for the YK1 strain (p < 0.05 or p < 0.01). The POMC mRNA level in the HFD group was increased markedly by A2-165 (p<0.05), DK3 (p<0.01), and DK11 (p < 0.05). In addition, the expression of the CCK2R gene was increased significantly by the DK3 (p < 0.05) strain, but not the other treatments.

### 3.9 *F. prausnitzii* treatment modulated the intestinal microbiota in HFD-induced obesity mice

**Figure 8:**
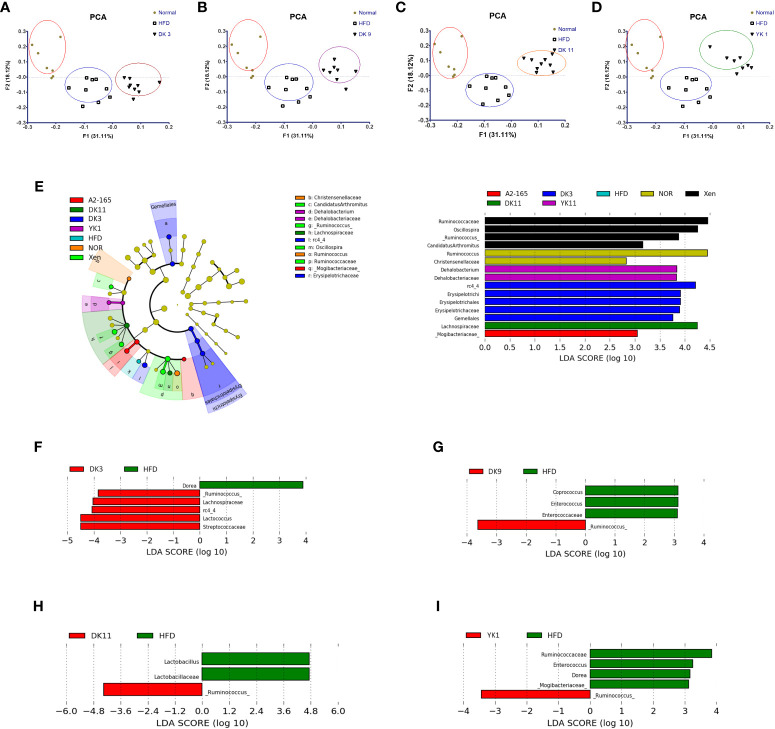
Effects of *F. prausnitzii* strains on gut microbiota composition. The gut microbiota was determined by 16S rRNA gene analysis of fecal samples from the mice. **(A–D)** Principal component analysis (PCA) of gut microbiota metagenomic samples. **(E)** Cladogram generated by the LEfSe analysis. The LEfSe plot shows enriched bacteria in all phenotypic categories. **(F–I)** LEfSe was used to identify bacteria represented differentially between DK3 and HFD **(F)**, DK9 and HFD **(G)**, DK11 and HFD **(H)**, and YK1 and HFD **(I)**. Only taxa meeting an LDA score threshold of two are listed.

Several recent studies have provided significant evidence to reveal a strong association between obesity and gut microbiota (36). 16S rRNA gene analysis of fecal samples from the mice was performed to explore changes to the gut microbiota after *F. prausnitzii* administration on HFD-fed obesity mice. The bacterial composition was examined by taxonomy-based analysis. The results showed that compared to normal low-fat diet food mice, HFD induced a significant change in the populations of the intestinal microbiota (**Figure 8**).

First, principal component analysis (PCA) was performed. **Figures 8A–D** shows the PCA results of the different treated groups. The results showed the different clustering positions of the mice within and across different treatment ways. The fecal samples of the mice with HFD clustered together, whereas most of the samples from the normal chow-diet-fed mice comprised another group. This result indicates that HFD feeding could induce differences in the gut microbiota compared to the normal group. In particular, distinct segregation of the gut microbial communities was observed in the DK3, DK9, DK11, and YK1 groups compared to the normal and HFD groups (**Figures 8A–D**). Unlike with four *F. prausnitzii* groups, the gut microbiota communities of A2-165 were not significantly different with normal and HFD groups (**Figure S4**).

Further analysis of the 16S rRNA sequencing data showed that the relative abundance of the gut microbial taxa differed among the experimental groups. The relative abundance of DK3, DK9, DK11, and YK1 was assessed at the genus level to identify the specific taxa related to selected strains supplementation (**Figure S5**). At the genus level, the relative abundances of *Ruminococcus* and *Lactococcus* were suppressed significantly in the HFD group compared to the normal chow diet group (p < 0.01). The *F. prausnitzii* strains could increase the abundance of *Ruminococcus, rc4-4, Parabacteroides, Lactococcus*, and *Bacteroides* significantly (p < 0.05 or p < 0.01). Furthermore, the increased proportions of sequences assigned to *Enterococcus* were significantly observed in the HFD group (p<0.05). In addition to *Dehalobacterium*, all the treatment groups significantly suppressed the increase in *Enterococcus* and *Coprococcus* (p<0.01). DK3, DK9, and DK11 markedly decreased the *Dehalobacterium* abundance (p<0.05). Overall, these results showed that administration of the selected strains *F. prausnitzii* could modulate the changes in these relative abundances of the gut microbiota induced by HFD-induced obesity.

### 3.10 Correlation between gut microbiota and obesity−related parameters

**Figure 9:**
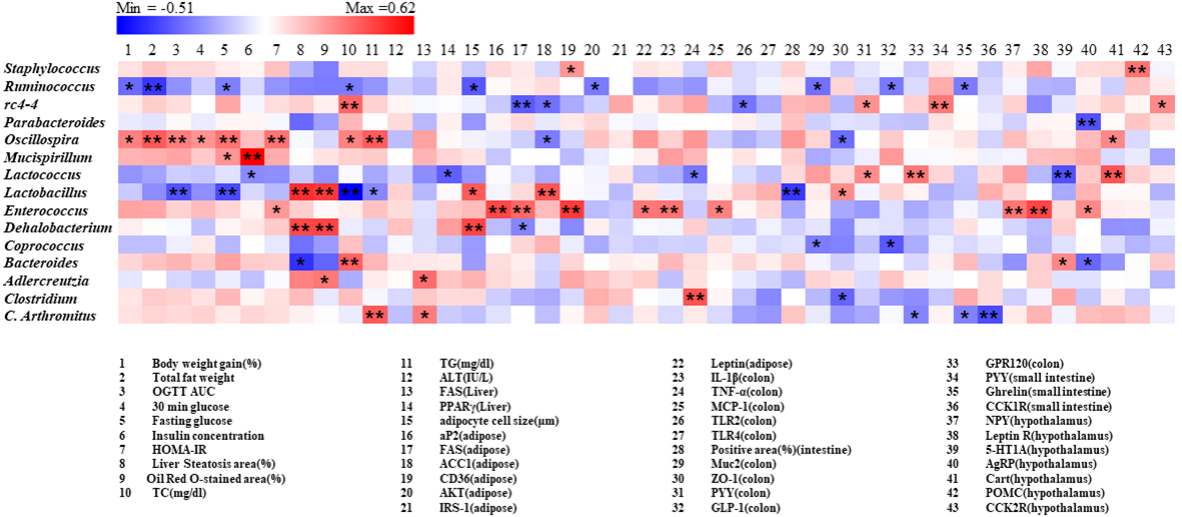
Correlation between gut microbiota and obesity related parameters. Pearson correlation results were used for heatmap establishment, with red indicating a positive correlation and blue indicating a negative correlation. “*” Denotes adjusted p<0.05 and “**” adjusted p<0.01.

A correlation matrix was established using Pearson’s correlation coefficient to completely investigate the relationships between levels of obesity biomarkers and abundance of gut microbiota (**Figure 9**). Intriguingly *Oscillospira* was positively correlated with body weight, fat weight, glucose metabolism related parameters, whereas *ruminococcus* showed negative correlation. On the other hand, *Lactobacillus* and *Dehalobacterium* were positively related with steatosis area (%) and Oil Red O-stained area (%) in liver, adipocyte cell size (μm) in adipose. Furthermore, it was indicated that rc4-4 shows a significant positive association with PYY levels in both colon and small intestine tissues, as well as a negative correlation with serum TC levels. Additionally, rc4-4 showed significant negative correlations with FAS and ACC1 levels in adipose tissues, and TLR2 levels in the colon.

## 4 Discussion

Obesity is linked to a number of complications, including specific anomalies in metabolism, such as hepatic steatosis, insulin resistance, hyperlipidemia, and some cancers (37, 38). Recent evidence has shown that probiotics have the potential to reduce obesity and improve metabolic parameters (39). *F. prausnitzii* is a major member of the gut microbiota in healthy adults. Accumulating evidence supports the health-promoting effects of *F. prausnitzii* (40), e.g., several metabolic-related parameters were improved in overweight and obese human subjects (22). A recent research suggested that the quantity of fecal *F. prausnitzii* is low in obese mice, and the abundance of *F. prausnitzii* could be increased by anti-obesity agents (41). Chronic HFD intake leads to obesity and hyperinsulinemia, hyperglycemia, and hypertension in rodents (42). Therefore, the HFD-induced mice model of obesity was used to compare the effect of pre-selected strains of *F. prausnitzii*.

In comparison to the normal group, the HFD group considerably outperformed it in terms of body weight gain, energy efficiency, calorie intake, weights of subcutaneous fat, epididymal fat, and mesenteric fat, as well as serum levels of TG and TC. The selected strains of *F. prausnitzii*, DK3, DK9, DK11, and YK1, reduced the body, liver, fat weight, and caloric intake compared to the HFD-fed animals. The fat reduction and lean mass increase showed that the *F. prausnitzii* treatment altered the body composition. In particular, only YK1 decreased the TC and TG serum levels compared to the other strains. Fat loss is related directly to glucose tolerance and insulin resistance (9). The four *F. prausnitzii* strains improved serum insulin and HOMA-IR. Moreover, DK11 and YK1 dramatically ameliorated the glucose tolerance rather than other strains. Therefore, these findings suggest that the HFD-induced obesity and associated metabolic abnormalities were improved by the selected *F. prausnitzii* strains.

The hepatic injury is associated with HFD-induced obesity (43). Probiotics have been found in numerous trials to enhance liver function (44). The disturbance of the glucose and lipid metabolism in the liver is linked to the pathogenesis of obesity, hepatic steatosis, and whole-body insulin resistance. FAS, ACC, and SREBP1 play essential roles in lipogenesis. FAS in the liver is a part of the lipogenic pathway (45), and SREBP1 mediates the induction of hepatic lipogenesis by insulin and glucose alteration (46). The *F. prausnitzi*i strains suppress hepatic lipogenic gene and protein expression, including FAS, ACC1, and SREBP1. These results are in accordance with the findings on biochemical index and histopathological staining. In particular, SREBP1c was significantly lower in DK11 than in the XEN group. In contrast, this value was much lower in the YK1 group than in the XEN and A2-165 groups. GLUT2 is the main glucose transporter in the hepatocytes of rodents and humans (47). As a direct target of PPARγ, hepatic GLUT2 aids in the transport of glucose into the liver (48). *Khiet Y. Trinh* et al. reported that increased-hepatic gluconeogenesis in obesity and T2DM is associated with insulin resistance, and the up-regulation of glucose-6-phosphatase (G6Pase) is crucial for the hepatic synthesis of glucose (49). Moreover, this study showed that *F. prausnitzii* stains, particularly DK9, reverse the levels of gluconeogenesis markers affected by the HFD treatment. Concomitantly with these data, a significant increase in liver weight and histological changes Page 13in hepatosteatosis was found, including hepatic steatosis area Page 13and marked oil droplets in HFD treated mice. In contrast, the *F. prausnitzii* strains reversed these elevations in keeping with the result from the mRNA levels of lipogenesis and gluconeogenesis markers.

Obesity leads to adipocyte hypertrophy and adipose tissue dysfunction. Adipocyte hypertrophy is the major mechanism for expanding the adipose tissue, and adipose tissue dysfunction leads to obesity (50). The size of the adipocytes and the adipose mRNA expression of the genes associated with insulin resistance were measured to clarify the mechanism of the *F. prausnitzii* strains on HFD-induced obesity. Adipocyte hypertrophy was identified as a major cause of adipose tissue expansion (51). The current investigation discovered that the *F. prausnitzii* strains decreased adipocyte size significantly in mesenteric fat tissue. Some studies have suggested that adipogenesis and lipolysis are complex processes involving several transcription factors (52), such as adipogenic differentiation-related genes aP2, lipogenesis genes FAS, ACC1, LDL, and insulin metabolism-related genes CD36, AKT, IRS, adiponectin, and leptin (53–56). In addition, the expression of the aP2, FAS, LDL, CD36, and leptin genes in the HFD-diet group was significantly higher than in the normal group. By contrast, the adiponectin mRNA level was lower than the normal group. aP2 is a lipid-binding protein that is upregulated during adipocyte differentiation, and the levels of circulating aP2 are dramatically elevated in dietary and genetic models of obesity (53). PCR showed that aP2 gene expression of adipose tissues is reduced by the treatment of *F. prausnitzii* in mice versus the HFD control. In the present study, gene over-expression of the lipogenesis markers, such as FAS, ACC1, and LDL, were significantly reduced by the *F. prausnitzii* strains in the adipose tissue. Therefore, *F. prausnitzii* strains potentially inhibited lipogenesis in adipose tissue, decreasing adipose accumulation in mice, which is consistent with fat mass reduction.

Leptin is mainly produced by adipose tissues, leading to insulin resistance development (57). CD36 is also associated with insulin resistance via modulation of lipid uptake (54). Of note, a significant expression reduction in the insulin resistance-related genes involved in *F. prausnitzii*-treated mice in accordance with the result of HOMA-IR. Furthermore, IRS-1 expression was decreased in type 2 diabetes and obesity subjects, and low IRS-1 expression causes a decrease in insulin-stimulated glucose uptake (58). Inhibition of Akt may cause insulin resistance because Akt is a major regulator of insulin action in muscle, fat, and liver (55). Adiponectin has a direct insulin-sensitizing action (59). Moreover, replenishing adiponectin reduced the insulin resistance and hypertriglyceridemia brought on by the HFD (56). Therefore, the reversal of the markers mentioned above by the *F. prausnitzii* strains inhibited adipogenic differentiation and lipogenesis and improved insulin resistance.

Increased levels of inflammatory cytokines contribute to the development of insulin resistance and obesity because this is linked to low-grade chronic inflammation (60). Previous studies reported that *F. prausnitzii* regulates the anti-inflammatory and pro-inflammatory cytokines to improve the inflammatory environment in the intestine (61). Pro-inflammatory cytokines such IL-1, IL-6, TNF-α, pro-inflammatory chemokine MCP-1, and toll-like receptors (TLR2 and TLR4) may be produced in different intestinal regions as a result of an HFD. These ultimately brought on the low-grade inflammation linked to metabolic diseases like insulin resistance, obesity, and others (39). Consistent with these findings, MCP-1, TNF-α, IL-6, IL-1β, TLR2, and TLR4 gene expression levels were substantially greater in the HFD group than in the normal group. Intestinal epithelial TLR signaling has a unique pathogenic function, according to *Everard* et al. TLRs abnormalities induce dysbiosis and predispose the individual to metabolic diseases (62). Therefore, TLRs play a crucial role in preserving intestinal and microbial homeostasis and contribute to the bowel inflammation brought on by the HFD and the ensuing metabolic abnormalities. The TLR2 and TLR4 gene expression was measured in the intestinal tissues to determine if *F. prausnitzii* affects the intestinal epithelial TLR signaling pathway. Based on the results, *F. prausnitzii* down regulated the gene expression of intestinal TLR2 and TLR4, which modulated the innate immunity of the intestinal epithelial cells. In accordance with these findings, these results demonstrated that the four *F. prausnitzii* strains had the ability to decrease the intestinal gene expression of IL-1, IL-6, and MCP-1.

The protective activity against an HFD-induced insult to intestinal integrity was evaluated because of the selected *F. prausnitzii* strains have anti-inflammatory characteristics. The anti-inflammatory process restored the gut barrier integrity in diet-induced obesity mice (63). AB staining of the mice intestine indicated that *F. prausnitzii* strains restored the damaged gut integrity caused by HFD. Goblet cells respond to microbial products to bolster the mucosal defense, which plays a key role in the host immunity and secretes mucin (64). Laura Wrzosek et al. reported that *F. prausnitzii* improves the establishment of epithelial homeostasis by modifying goblet cells and mucin glycosylation (65). The results showed that *F. prausnitzii* strains-increased goblet cells produce more mucin, thickening the intestinal barrier. The tight junction proteins modulate the intestinal permeability, including ZOs, JAMs, occludin, and claudins. These junction proteins, which firmly join epithelial cells, are essential for controlling the effectiveness of the intestinal barrier (66). Previous studies suggested that HFD feeding induced various intestinal physiopathological dysbioses, such as alteration of the gut integrity and intestinal permeability (67). These results showed that *F. prausnitzii* is important in improving the gut barrier function. The data suggested that it could be a key mechanism through which *F. prausnitzii* restored gut barrier function and lessened gut permeability. In general, the gut-brain axis reflects the interaction that is present between the gastrointestinal system and the brain. In response to food consumption, the brain gets neuronal and endocrine inputs from the gut, which are combined with signals from other organs to coordinate physiological responses (26). The hypothalamic nuclei are major brain integrating centers. In this study, the hypothalamus, colonic and small intestine tissue were used to provide evidence of the gut–brain cross talk involved in regulating food intake. PYY is a peptide that enteroendocrine L cells release and plays a role in controlling appetite. Evidence has shown that PYY may affect body weight by reducing appetite and raising energy expenditure (68). L cells in gastrointestinal produce the incretin hormone GLP-1, exerts many biological functions, such as inducing satiety and slowing gastric emptying. The L cells of distal gut release PYY and GLP-1 after nutrient ingestion to reduce appetite (69). This study showed that *F. prausnitzii* strains could modulate the gene expression of PYY and GLP-1 in the jejunum and colon. Recently, some studies reported that several orphan G protein-coupled receptors (GPCRs), GPR41, GPR43, and GPR120, possess potential as fresh drug targets for disorders of metabolism, including T2D and obesity (70). Intestinal GPR120 is widely expressed and mediates GLP-1 secretion (71). Through the gut-brain neurological network, GPR41 expression in intestinal L cells, which release GLP-1 and PYY, also improves insulin sensitivity (70). GRP43 regulates appetite and PYY secretion and is expressed in enteroendocrine L cells (72). All selected strains of *F. prausnitzii* were used to treat the HFD-fed mice, and this treatment increased the mRNA levels of GPR41, GPR43 and GPR120 thereby affecting hormone secretion in the colon. According to reports, *F. prausnitzii* is one of the main butyrate producers in the intestine (73). Interestingly, when the butyrate binds to its receptors, GPR41 and GPR43, on L cells promote the synthesis of PYY and GLP-1 (74, 75).

Cholecystokinin (CCK) is a brain–gut peptide (76). The postprandial inhibition of stomach emptying and inhibition of colonic transit are the CCK motor effects. At the same time, CCK can activate direct vagal afferent fibers and modify the vagal mechanosensitive fibers to gastric and duodenal loads response properties (77). Currently study, a significant downregulation of the small intestine gene expression of CCK and CCK1R was found in HFD-fed mice. In addition, exposure of the HFD group to YK1 increased the mRNA levels of GIP in the jejunum, which can interact with GLP-1 to regulate energy absorption and food intake (78). In contrast, administration of DK3 and DK11 to the HFD group reduced the ghrelin gene expression in the jejunum. As seen in the current investigation, reversing the production of the aforementioned brain-gut peptide in the HFD-fed mice with *F. prausnitzii* supplementation may enhance ghrelin secretion and suppress appetite.

Furthermore, the *F. prausnitzii* strains modulated the mRNA expression of hormone secretion in the hypothalamus, and evidence of the gut–brain cross talk in controlling food intake was found. GHSR is a specific receptor of Ghrelin and exerts several physiological effects (79). In addition, NPY/AgRP neurons were activated by ghrelin (80). Thus, the levels of Ghrelin in the jejunum, as well as GHSR, NPY, and AgRP in the hypothalamus, are reduced significantly after administering the *F. prausnitzii* strains. The endogenous hypothalamic serotonin (5-HT) has been reported to be linked with the processes of satiety during meals and the final stage of post-meal satiety (81). In the present study, the *F. prausnitzii* strains modulated the mRNA expression of the 5-HT1A and 5-HT1B in the hypothalamus, which are the most directly 5-HT receptors implicated in feeding control. GIPR is expressed in the hypothalamus, which helps control food intake. Hypothalamic Gipr^+^ neuron activation inhibits food intake in mice. In addition, the brain as a target organ is based on the GIP pharmacology (82). In the present study, *F. prausnitzii* strains increased the gene expression of GIPR in the hypothalamus and GIP in the jejunum. Hence, *F. prausnitzii* strains can simultaneously affect the secretion of appetite-related hormones in the small intestine and hypothalamus. Furthermore, the HFD group’s exposure to the *F. prausnitzii* strains improved the expression of the Cart and POMC genes, which are anorexia peptides in hypothalamus.

Numerous evidence suggests a close association between obesity and gut microbiota, whereas probiotic therapy reduces obesity and intestinal dysbiosis by regulating the gut microbiota (39). 16S rRNA sequencing analysis of fecal samples was performed to investigate whether and how *F. prausnitzii* strains influence the gut microbiota. PCA analysis showed that *F. prausnitzii* affects the composition of the gut microbiota underwent clear modifications in the gut microbial structure compared to HFD-diet mice. As expected, *F. prausnitzii* affects the gut microbiota composition by increasing the presence of genus *Ruminococcus*, *rc4-4*, *Parabacteroides*, *Lactococcus*, and *Bacteroides* while decreasing *Dehalobacterium*, *Enterococcus*, and *Coprococcus* in HFD-fed mice. Evidence suggests that *Oscillospira* is associated with leanness or lower body mass index (BMI) for both adults and children. The increased abundance of *Oscillospira* was recently found to correlate with the health subjects (83). By supplementing *F. prausnitzii*, the *Oscillospira* abundance was increased significantly in the HFD group, which may explain part of the beneficial effects of the *F. prausnitzii* strains. As common probiotics, *Lactococcus* spp. are used to improve human and animal health. Recent studies have linked *Lactococcus lactis* to insulin resistance and systemic inflammation, exerting an anti-obesity effect (84). Herein, a higher abundance of *Lactococcus* was found in all *F. prausnitzii* strain treated groups than in the HFD group, which may assist in weight control. Gut *Parabacteroides* are important members of the human gut microbiota and have a lower abundance in those who have nonalcoholic fatty liver disease and obesity (85). *Parabacteroides* also has metabolic benefits in decreasing hepatic steatosis, hyperglycemia and weight gain (86). Obese individuals are associated with decreased *Bacteroides*, and an increased abundance of *Bacteroides* was recently found to correlate with benefits to health (35 ). In addition, the abundance of *Bacteroides* has been implicated in the appearance of diabetes-related auto-antibodies, according to reports (87). YK1 strains increased the abundance of *Parabacteroides*, and YK1 and DK11 increased the *Bacteroides* abundance, suggesting that these strains alleviate hepatic steatosis and metabolic abnormal. *Dehalobacterium* showed a significant increase in HFD-induced obesity mice (88). Additionally, in a prior clinical investigation, compared to participants who were non-obese subjects, obese subjects had greater *Coprococcus* levels (89). In the present study, *F. prausnitzii* strains, DK3, DK9, and DK11, obviously changed the gut microbial communities at the genus level by decreasing the population of *Dehalobacterium* and *Coprococcus*. Previous studies have shown inconsistent results for the ratio of *Lactobacillus* and *F. prausnitzii*. For example, a recent study showed that consuming *Lactobacillus johnsonii La1* decreased the *F. prausnitzii* levels in healthy subjects (90). Meanwhile, potato fiber (FiberBind 400) improved Lactobacillus survival, while decreasing the abundance of *F. prausnitzii* (91). However, the Lactobacillus was increased while *F. prausnitzii* was decreased in the patients with inflammatory bowel disease as compared to healthy control (92). Taken together, these findings suggest that probiotics have strain-specific effects (61) and further research is needed to understand the underlying mechanisms.

While a prior study demonstrated that one strain of *F. prausnitzii* (ATCC 27766) has anti-obesity effects in the same animal model (22), our study used four different *F. prausnitzii* strains for a thorough effective comparison, all of which were newly isolated from the human gut. Even within the same species, different bacterial strains can exhibit varying degrees of differences in properties (93). In our previous study, we investigated how different strains of *Akkermansia muciniphila* (*A. muciniphila*) improve metabolic disorders in HFD-induced mice, and found that even within the same species, different bacterial strains can elicit different responses from the host (39). In the present study, the three *F. prausnitzii* strains increased the abundances of Ruminococcus, rc4-4, Lactococus and Oscillospira in different degrees, while DK11 decreased the relative abundance of Lactobacillus. The exact cause is still not fully clear, but differences in the gut microbiome emphasize the importance of our research on the distinctions between various strains. In addition, it has long been a conventional practice to study the interaction between microbes and hosts using mouse models. However, phylogenetic and metagenomics research has revealed that while mouse fecal, caecal, and human fecal samples have a large degree of overlap, they also differ significantly in abundance (94). Consequently, future studies should consider this limitation to accurately demonstrate the impact on humans.

It is worth noting that some specific gut microbiota is strongly correlated with obesity and its related metabolic disorders. For instance, *Oscillospira* exhibited a strong positive correlation with many obesity-related parameters, while the treatment with *F. prausnitzii* strains appeared to show a decreasing trend in its relative abundance. Rc4-4 displayed a strong negative correlation with the RNA expression of adipose FAS and ACC1 levels, as well as colon TLR2 levels. These correlation findings provide valuable insights into the associations between obesity biomarkers and the gut microbiota.

Overall, YK1 was found to be more effective in regulating glucose homeostasis, suppressing triglyceride levels, and inhibiting lipogenesis, as well as regulating hormone secretion in the hypothalamus. DK11 was found to have a better impact on glucose homeostasis, triglycerides, and the function of the intestinal barrier, as well as hormone secretion in the colon, but was less effective in suppressing lipogenesis compared to other strains. DK9 was found to have less efficiency in preventing liver damage, but had a greater impact on the intestinal tract. DK3 was found to have better effects in suppressing lipogenesis and regulating hormone secretion in the hypothalamus. Based on these results, it is speculated that YK1 and DK3 have a greater impact on the hypothalamus, with YK1 showing more significant inhibition of appetite-promoting hormones and DK3 releasing more appetite-suppressing hormones. Meanwhile, DK11 and DK9 have a more significant impact on the intestinal tract. DK11 may primarily affect hormone secretion in the colon and DK9 may primarily affect hormone secretion in the jejunum.

## 5 Conclusions

In conclusion, four novel human intestine-derived *F. prausnitzii* strains, DK3, DK9, DK11, and YK1, ameliorated HFD-induced obesity and related metabolic disorders and had better efficiency than type strain A2-165 and a comparison group XEN. These *F. prausnitzii* strains could inhibit low-grade inflammation, restore the gut integrity, improve hepatic injury and insulin resistance, modulate hormone secretion, regulate appetite, and inhibit lipogenesis by affecting the gut–brain axis. These observations show that four *F. prausnitzii* strains exerted functional differences surpassing the type strain. Moreover, *F. prausnitzii* makes a favorable contribution to the gut ecosystem due to a significant relationship between *F. prausnitzii* supplementation and gut microbiota composition. Therefore, all results provide a rationale for developing a treatment that uses different strains of *F. prausnitzii* to prevent or treat obesity and its associated metabolic disorders.

## 6 Institutional review board statement

This research was approved by the Institutional Review Board of Dongguk University (approval number: IRB# 2018-06-001-012).

## Data availability statement

The datasets presented in this study can be found in online repositories. The names of the repository/repositories and accession number(s) can be found below: https://www.ncbi.nlm.nih.gov/,PRJNA885263.

## Ethics statement

The studies involving humans were approved by the Institutional Review Board of Dongguk University, Ilsan Hospital (IRB# 2018-06-001-012). The studies were conducted in accordance with the local legislation and institutional requirements. The participants provided their written informed consent to participate in this study. The animal study was approved by All experiments were approved by the institutional animal care and use committee (IACUC) of the Dongguk University (2020-11208) and conducted according to the guidelines of the National Research Council (Guide for the Care and Use of Laboratory Animals, 2011). The study was conducted in accordance with the local legislation and institutional requirements.

## Author contributions

Conceptualization, HK and J-GS. Methodology, MY, Joo-HS, DL, and Ji-HS. Formal analysis, MY. Writing - original draft preparation, MY and J-HW. Visualization, MY and J-HW. Supervision, HK, JGS, XS, and Y-DN. Funding acquisition, HK, JGS, and Y-DN. All authors contributed to the article and approved the submitted version.
