# Supplementary material for: Pharmaceutical efficacy of novel human-origin Faecalibacterium prausnitzii strains on high-fat-diet-induced obesity and associated metabolic disorders in mice
Source: Front Endocrinol (Lausanne). 2023 Aug 29;14:1220044. doi: 10.3389/fendo.2023.1220044 (PMC10497875; doi:10.3389/fendo.2023.1220044)
Supplement: Supplementary file 1 [file Presentation_1.pdf]

Pharmaceutical efficacy of novel human-origin *Faecalibacterium prausnitzii* strains on high-fat-diet-induced obesity and associ-ated metabolic disorders in mice

Meng Yang, Jing-Hua Wang, Joo-Hyun Shin, Dokyung Lee, Sang-Nam Lee, Jae-Gu Seo, Ji-Hee Shin, Young-Do Nam*, Hojun Kim*, Xiaomin Sun*

Supplementary Material

Supplementary Figures

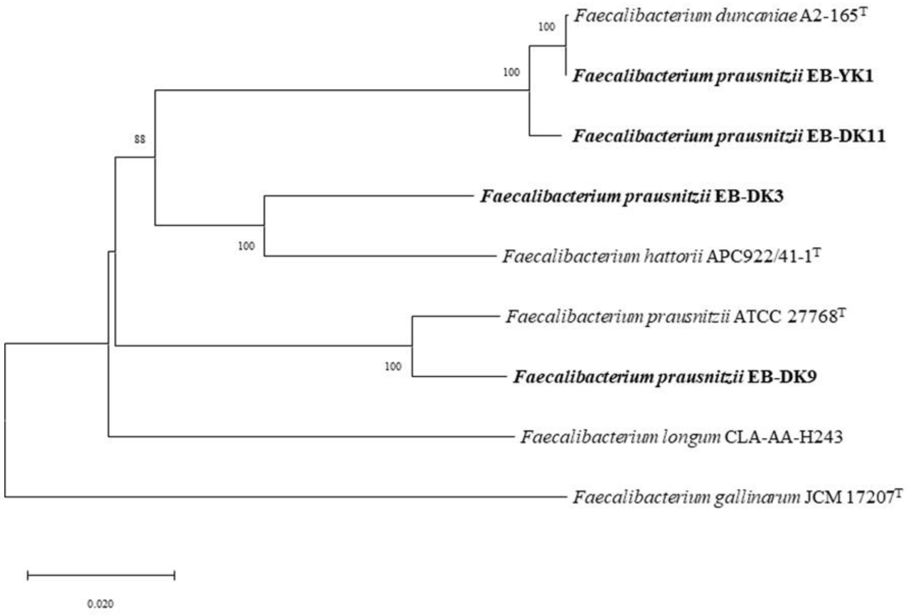

Supplementary Figure 1. Genetic diversity of *F. prausnitzii* strains. Phylogenetic trees of *Faecalibacterium* sp. were generated based on seven MLST loci using the neighbor-joining method in MEGA-X software with the Kimura 2-parameter model. A total of 1,000 bootstrap replicates were performed. The scale bar represents the number of substitutions per site.

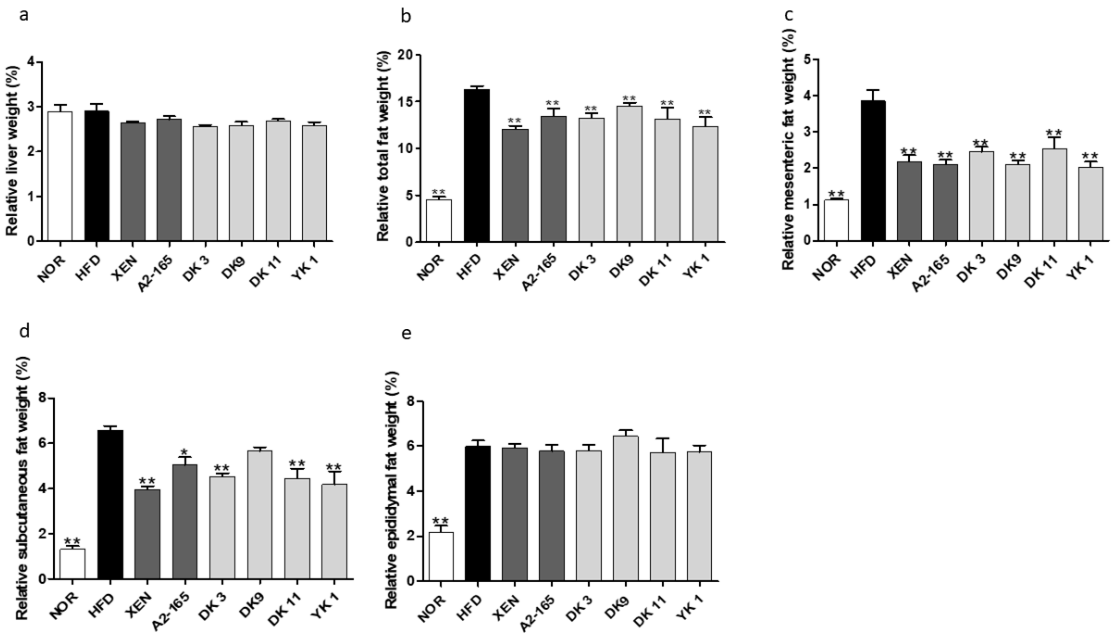

Supplementary Figure 2. Tissue mass as relative to body weight. (a) Relative liver weight (%). (b) Relative total fat weight (%). (c) Relative mesenteric fat weight (%). (d) Relative subcutaneous fat weight (%). (e) Relative epididymal fat weight (%). Data are represented as the mean±SEM (n=9). The statistics were analyzed by one-way ANOVA. * $p < 0.05$ and ** $p < 0.01$ versus the HFD group.

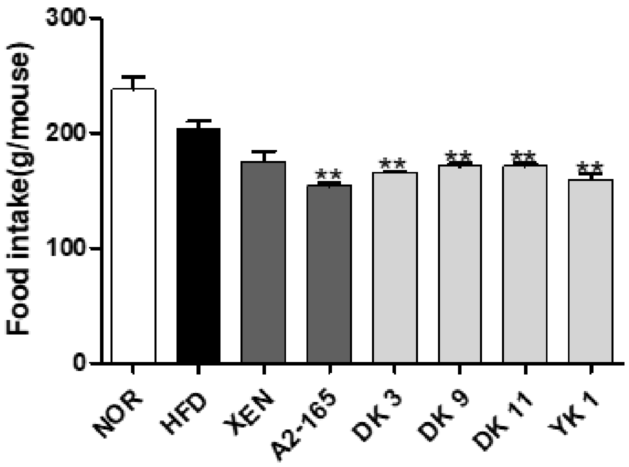

Supplementary Figure 3. *F. prausnitzii* modulated the food intake in high-fat-diet mice. Body weight was measured weekly. Data are represented as the mean ± SEM (n = 9). Statistics were analyzed by one-way ANOVA. *p $<$ 0.05 and **p $<$ 0.01 versus the HFD group.

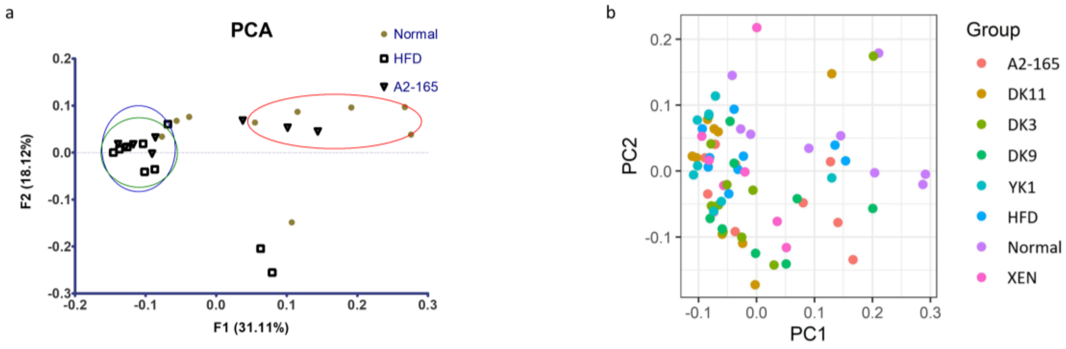

Supplementary Figure 4. Comparison of gut microbiota composition. The principal component analysis (PCA) among three groups (Normal, HFD, and A2-165 group) (a) and eight groups (A2-165, FDDK11, FDDK3, FDDK9, FDYYK1, HFD, Normal, and XEN) (b).

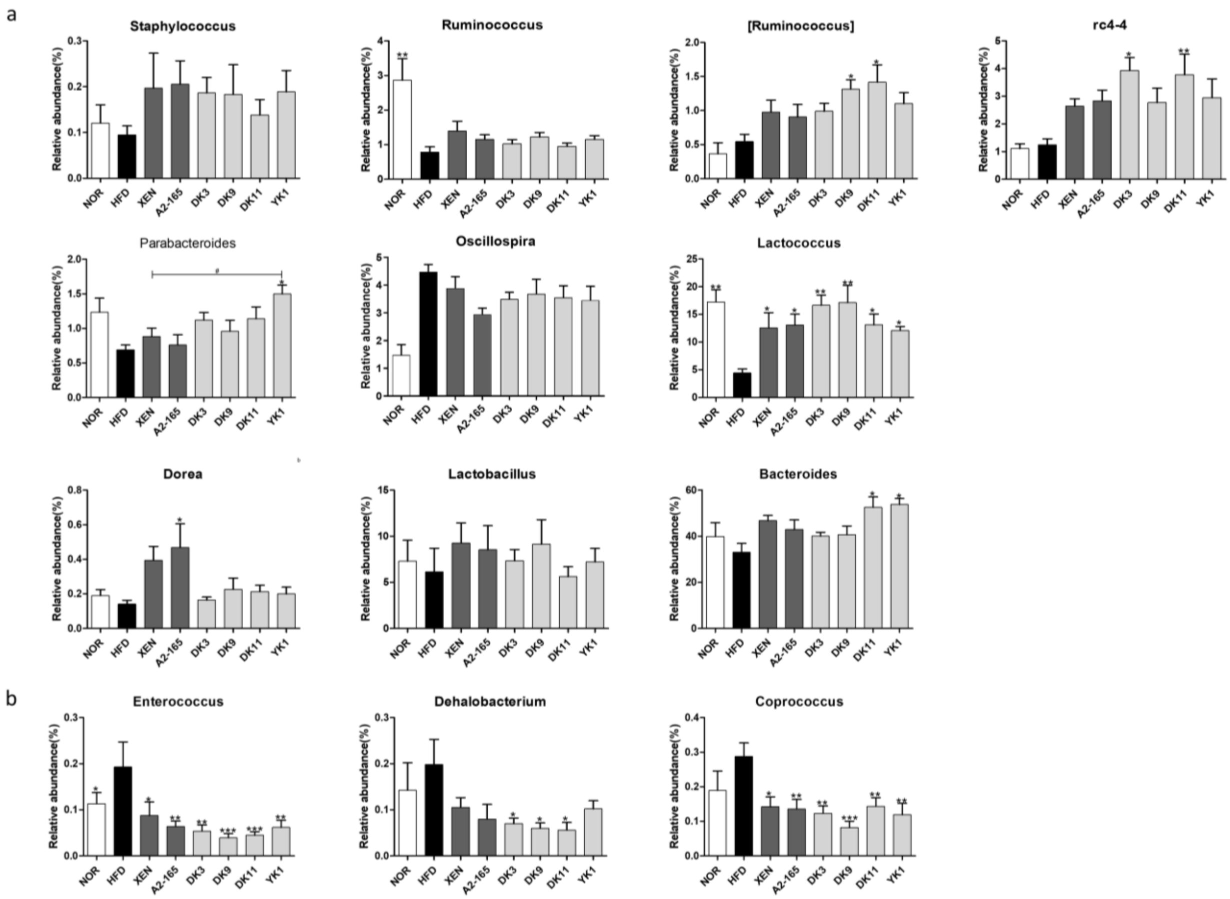

Supplementary Figure 5. *F. prausnitzii* modulated the composition of gut microbiota. Relative abundance of the gut bacterial genus-level detected in fecal samples. Data are represented as the mean ± SEM (n = 9). Statistics were analyzed by one-way ANOVA. *p＜0.05, **p＜0.01, and ***p＜0.001 versus the HFD group.

Supplementary Table

Supplementary Table 1. *F. prausnitzii* strains improved lipid metabolism parameter of the HFD mice.

Gene	Primer Sequence (5'–3')	Gene	Primer Sequence (5'–3')
ACC1	F: CCTCCGTCAGCTCAGATACA<br>R: TTTACTAGGTGCAAGCCAGACA	PYY	F: CGGCAGCGGTATGGAAAAA<br>R: TGTGAAGAGCAGTTTGGAGAACA
FAS	F: AGGGGTCGACCTGGTCCTCA<br>R: GCCATGCCCAGAGGGTGGTT	GLP-1	F: GGCACATTCACCAGCGACTAC<br>R: CAATGGCGACTTCTTCTGGG
SREBP1c	F: GGAGCCATGGATTGCACATT<br>R: GGCCCGGGAAGTCACTGT	GPR120	F: GTGCCGGGACTGGTCATTGTG<br>R: TTGTTGGGACACTCGGATCTGG
GLUT2	F: GGCTAATTTCAGGACTGGTT<br>R: TTTCTTTGCCCTGACTTCCT	GPR43	F: ACAGTGGAGGGGACCAAGAT<br>R: GGGGACTCTCTACTCGGTGA
G6Pase	F: GACTCCCAGGACTGGTTCAT<br>R: GGGCGTTGTCCAAACAGAAT	GPR41	F: GGGGTCGATACAAGAGT<br>R: CTGGCGGAGCTACGTGCT
PPARγ	F: GCCCTTTGGTGACTTTATGGA<br>R: GCAGCAGGTTGTCTTGGATG	CCK	F: GCACTGCTAGCGCGATACATC<br>R: CCAGGCTCTGCAGGTTCTTAAG
Ap2	F: AGTGAAAACTTCGATGATTACATGAA<br>R: GCCTGCCACTTTCCTTGTG	Ghrelin	F: CAGAAAGCCCAGCAGAGAAA<br>R: GAAGGGAGCATTGAACCTGA
LDL	F: TTGCCCTAAGGACCCCTGAA<br>R: ACAGAGTCTGCTAATCCAGGAAT	GIP	F: GACTTCGTGAACTGGCTGCT<br>R: TTGTCCTCCTTTCCCTGAGA
CD36	F: TTGTACCTATACTGTGGCTAAATGAGA<br>R: CTTGTGTTTTGAACATTTCTGCTT	CCK1R	F: AGCAGTCCTGGCAAACATTCCTGC<br>R: GCATGCGGATCACGCGCTTC
AKT	F: CATGAGGATCAGCTCGAACAGC<br>R: ACGGGCACATCAAGATAACGG	CCK2R	F: GCCGTTTCCTACCTCATGGGGGT<br>R: CCCTTGGTTTCGGACCCGGC
IRS1	F: TTTGAAGACCATAACCCACCA<br>R: ATTACACCAGTTCGTCCCTTTC	GHSR	F: GGACCAGAACCACAAACAGACA<br>R: CAGCAGAGGATGAAAGCAAACA
Adipnectin	F: GGCAGGAAAGGAGAACCTGG<br>R: AGCCTTGTCCTTCTTGAAGA	NPY	F: CAGAAAACGCCCCCAGAA<br>R: AAAAGTCGGGAGAACAAGTTTCATT
Leptin	F: GCCAGGCTGCCAGAATTG<br>R: CTGCCCCCCAGTTTGATG	Leptin R	F: GCCGGTGTGAGTTTTCAGTCA<br>R: CCTAAGGGTGGATCGGGTTT
IL-1β	F: TGCCACCTTTTGACAGTGATG<br>R: AAGGTCCACGGGAAAGACAC	5-HT1A	F: ACTCCCTGCTCAACCCAGTTATTTA<br>R: CACTCTTCCTCCACTTCTTCCTTCT
IL-6	F: CCTCTGGTCTTCTGGAGTACC<br>R: ACTCCTTCTGTGACTCCAGC	5-HT1B	F: GCGGTGGAGTATTCTGCTAAA<br>R: GAAGGGTGGCAACGAAATAGA
TNFα	F: GACCCTCACACTCAGATCATCTTCT<br>R: CCACTTGGTGGTTTGCTACGA	AgRP	F: CGGAGGTGCTAGATCCACAGA<br>R: AGGACTCGTGCAGCCTTACAC
MCP-1	F: AAGAGATCAGGGAGTTTGCT<br>R: CTGCCTCCATCAACCACTTT	Cart	F: CTGCAATTCTTTCCTCTTGAAGTG<br>R: GGGAATATGGGAACCGAAGGT
TLR2	F: AAGGAGGTGCGGACTGTTTC<br>R: GAGCCAAAGAGCTCGTAGC	GIPR	F: GCGTGCTCTACTGCTTCATCAAC<br>R: AACTTTCCAAGACCTCATCCCC
TLR4	F: CCTGATGACATTCCTTCTTCAAC<br>R: TTGTTTCAATTTCACACCTGGATAAA	POMC	F: TGCTTCAGACCTCCATAGATGTGT<br>R: GGATGCAAGCCAGCAGGTT
Occludin	F: ATGTCCGGCCGATGCTCTC<br>R: TTTGGCTGCTCTTGGGTCTGTAT	MUC2	F: CCATTGAGTTTGGGAACATGC<br>R: TTCGGCTCGGTGTTCAGAG
JAM-A	F: TCTCTTCACGTCTATGATCCTGG<br>R: TTTGATGGACTCGTTCTCGGG	ZO-1	F: TTTTTGACAGGGGGAGTGG<br>R: TGCTGCAGAGGTCAAAGTTCAAG
